# Haplotype-resolved genome of heterozygous African cassava cultivar TMEB117 *(Manihot esculenta)*

**DOI:** 10.1038/s41597-023-02800-0

**Published:** 2023-12-09

**Authors:** Michael Landi, Trushar Shah, Laurent Falquet, Adnan Niazi, Livia Stavolone, Erik Bongcam-Rudloff, Andreas Gisel

**Affiliations:** 1 Department of Animal Breeding and Genetics, Bioinformatics, Swedish University of Agricultural Sciences, Uppsala, Sweden; 2 International Institute of Tropical Agriculture, Nairobi, Kenya; 3 Department of Biology, University of Fribourg, Fribourg, Switzerland; 4 Swiss Institute of Bioinformatics, Lausanne, Switzerland; 5 International Institute of Tropical Agriculture, Ibadan, Nigeria; 6 Institute for Sustainable Plant Protection, Consiglio Nazionale delle Ricerche, Bari, Italy; 7 Institute for Biomedical Technologies, Consiglio Nazionale delle Ricerche, Bari, Italy

**Keywords:** Bioinformatics, Agriculture, Genome

## Abstract

Cassava (*Manihot esculenta Crantz*) is a vital tropical root crop providing essential dietary energy to over 800 million people in tropical and subtropical regions. As a climate-resilient crop, its significance grows as the human population expands. However, yield improvement faces challenges from biotic and abiotic stress and limited breeding. Advanced sequencing and assembly techniques enabled the generation of a highly accurate, nearly complete, haplotype-resolved genome of the African cassava cultivar TMEB117. It is the most accurate cassava genome sequence to date with a base-level accuracy of QV > 64, N50 > 35 Mbp, and 98.9% BUSCO completeness. Over 60% of the genome comprises repetitive elements. We predicted over 45,000 gene models for both haplotypes. This achievement offers valuable insights into the heterozygosity genome organization of the cassava genome, with improved accuracy, completeness, and phased genomes. Due to its high susceptibility to African Cassava Mosaic Virus (ACMV) infections compared to other cassava varieties, TMEB117 provides an ideal reference for studying virus resistance mechanisms, including epigenetic variations and smallRNA expressions.

## Background & Summary

Plants exhibit remarkable genetic diversity, often as a mosaic of different variants within a single individual. Crops like cassava, mango, and rubber tree are often highly heterozygous because of either outcrossing or clonal propagation^1–3^. Plants propagated clonally through methods such as stem cutting retain genetic variation, making it challenging to create high-quality reference genomes. Despite this challenge, the recent advancements in sequencing technologies have made it possible for researchers to explore the complex genomic architecture of these crops^4^. By uncovering and analyzing the genomic diversity of plants, we can fully harness their potential and facilitate innovations in the fields like breeding, agronomy, and food security.

Cassava (*Manihot esculenta Crantz*) is a vital crop for subsistence farmers in tropical and subtropical regions across the globe, providing a source of food and industrial purposes. Cassava is utilized to produce various products such as starch, bioethanol, and other bio-based products such as feed, medicine, cosmetics, and biopolymers^5^. Subsistence farmers in Africa prefer cultivating cassava as it yields substantial harvests under diverse environmental conditions^6^. Additionally, cassava roots have an ideal harvesting age and can be harvested flexibly, offering the benefits of a longer in-ground storage^7^. However, the crop faces pests, diseases, drought, weeds, and environmental factors that limit its productivity. Developing more resilient and productive cassava varieties through conventional breeding is time-consuming. Therefore, having a complete haplotype-resolved genome with high accuracy can be a valuable resource in cassava breeding and genomics.

Cassava has a haploid genome of about 750 Mbp, a highly heterozygous and repetitive plant genome^8^. Despite the use of various sequencing technologies over time, there are unresolved gaps in the genome. The reference genome AM560-2, derived from a Colombian cassava line MCol505, has undergone steady improvement over a decade and has had five major releases, with the current version being AM560-2 version 8^4^. While this reference genome benefits the cassava community, it does not capture the genetic diversity in African cassava cultivars grown by smallholder farmers due to its homozygous nature. Recently, attempts have been made to assemble genomes of African cassava lines such as TME3 and 60444, using a combination of Illumina short reads, PacBio long reads, bio-nano optical mapping, and chromatin conformation capture (Hi-C) sequence technologies producing assemblies of N50 of 98 and 117 Kbp. The assembled genomes had large contiguous assemblies but lacked haplotypic separation, containing multiple copies of duplicated sequences in the primary assembly^9^. The TME7 genome was assembled using a combination of Illumina, PacBio, and Hi-C sequencing technology to generate a contiguous genome assembly of N50 of approximately 320 Kbp. This genotype was successfully deduplicated and phased using Hi-C sequence data^10^. The most recent African cassava genotype to be assembled is TME204, which was phased using Hi-C technologies and PacBio high-fidelity (HiFi) sequencing reads, resulting in a highly contiguous assembly of N50 > 18 Mbp^11^. PacBio HiFi sequencing technology has proven effective in creating long and highly accurate reads for assembling complex genomes^12^. Recent studies demonstrate its potential in assembling high-quality plant genomes, including *Populus tomentosa* Carr, the 35.6 Gb California redwood genome and *Bletilla striata*^13–15^.

**Fig. 1:**
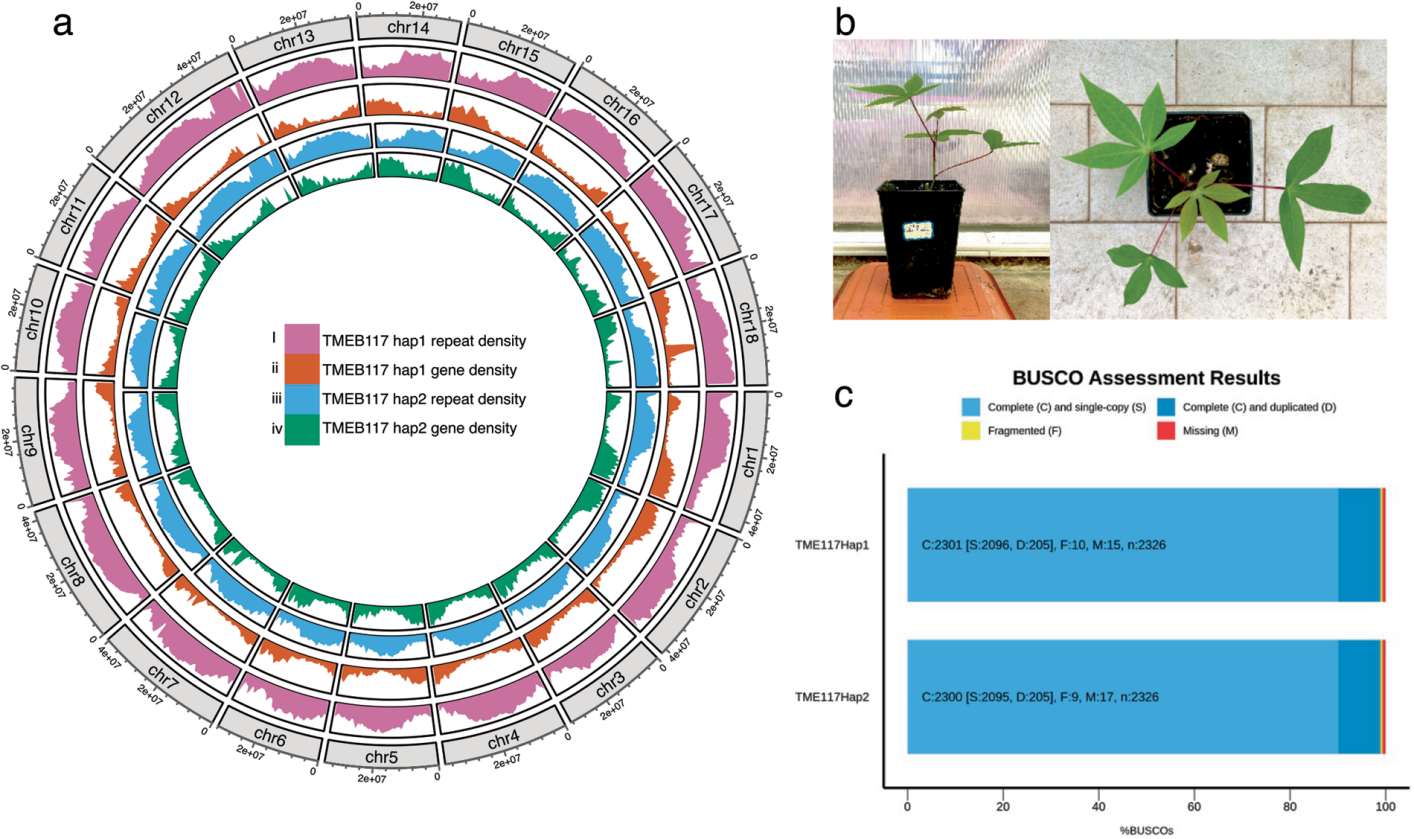
Overview of the cassava cultivar TMEB117 genome. (**a**) Circos plot displays repeat and gene densities for the two haplotypes visualized in 1 Mbp sliding windows. The tracks from the outer to inner show, (i) Repeat density for hap1 genome (ii) Gene density for hap1 genome (iii) Repeat density for hap2 genome (iv) Gene density for hap2 genome. (**b**) Cassava plant in a pot from the screen house. (**c**) BUSCO score of the TMEB117 genome.

In this study, we have generated a haplotype-resolved diploid assembly of TMEB117, a farmer-preferred cassava cultivar, using PacBio HiFi reads. TMEB117 (also called TME117, TME 117, and ISUNIKANKIYAN) is a Nigerian cassava landrace highly susceptible to African cassava mosaic virus (ACMV)^16^. This genotype served as a reference for ACMV studies^16^ and a high-resolution genome will pave the way for future investigations in epigenetics and small RNA expression analysis to learn more about the mechanisms of ACMV resistance in cassava to support future breeding programs. The TMEB117 hap1 assembled genome had a total size of 694 Mbp and hap2 665 Mbp with a contig N50 length of 18 Mbp (hap1) and 17 Mbp (hap2) (Table 1). These assembled haplotigs were further ordered and scaffolded using the TME204 reference genome to produce a chromosome-scaled genome for TMEB117 and enhanced contiguity with an improved N50 length exceeding 35 Mbp in both haplotypes (Table 1). The haplotype-specific annotations for TMEB117 hap1 and hap2 genomes resulted in 47,138 and 49,163 gene models, respectively. Within the TMEB117 hap 1 genome, a total of 442 Mbp (65.34%) was occupied by repetitive elements, whereas hap2, 408 Mbp (60.32%), encompassed by transposable elements. Evaluation of the final genome exhibited a high completeness of 98.9%, according to BUSCO^17^. The two haplotype genomes attained a high base-level accuracy of QV > 64. Furthermore, in the raw data, we detected reads that closely matched the entire genome of the fungus *Alternaria alternata*, despite the plants being healthy and showing no symptoms. We eliminated these contaminant reads and excluded them from the final assembly. The phased and annotated homologous chromosomes provide a comprehensive perspective of cassava’s heterozygous genome organization with improved accuracy and completeness at a haplotype-resolved level (Fig. 1a). These chromosome pairs are anticipated to be a valuable resource for cassava breeders and essential for functional analysis to characterize molecular mechanisms important agronomical.

**Table 1:** Assembly quality metrics generated by QUAST for the assembly produced by hifiasm before and after ordering and scaffolding.

Description	Contig-level assembly statistics hap1	Chromosome-level assembly statistics hap1	Contig-level assembly statistics hap2	Chromosome-level assembly statistics hap2
**Number of contigs**	362	299	159	96
**Number of contigs > = 25000 bp**	317	262	156	96
**Number of contigs > = 50000 bp**	100	52	103	45
**Largest contig (bp)**	43,434,407	51,416,241	35,731,055	50,183,282
**Total length (bp)**	693,971,781	693,957,521	664,959,903	664,966,403
**N50**	18,674,865	37,612,488	17,299,599	35,761,448
**N90**	9,202,869	32,042,468	6,549,198	30,936,428
**L50**	13	9	13	9
**L90**	33	17	33	17
**GC (%)**	37.81	37.81	37.62	37.62
**N’s per 100 Kbp**	0	0.49	0	0.53
**N’s**	0	3412	0	3492

## Methods

### Sampling, sequencing, sequence quality and contamination check

Cassava plants of the TMEB117 genotype, obtained from the International Institute of Tropical Agriculture (IITA) Genebank collection^18^, were grown in pots in a screen house (Fig. 1b). Third and fourth fully-expanded leaves of a potted plantlet hardened from *in vitro* culture were used for genomic DNA extraction using an optimized version of the CTAB (2% CTAB, 2% PVP-40, 20 mM Tris-HCl, pH 8.0, 1.4 M NaCl, 20 mM ethylenediaminetetraacetic acid) total nucleic acid extraction protocol as described by Carluccio, A. V. *et al*.^19^. After RNase treatment, the resulting DNA was cleaned using the Genomic DNA clean and concentrator kit (Zymo Research) according to the manufacturer’s instructions. We improved the gene annotation step by utilizing RNA data from another project, previously extracted from leaves of approximately two-month-old greenhouse plants at IITA Ibadan. The extraction process employed a combination of CTAB and spin column-based purification methods. The total RNA was then sequenced using the Illumina HiSeq. 2500 with a paired-end 2 × 100 cycle approach. The total DNA sample was sequenced with PacBio technology (PacBio Sequel II platform) using two SMRT cells. In the first cell, 875,686 reads and 1,163,062 reads in the second cell were generated. The raw sequence reads obtained from the two SMRT cells were combined. Fastp version 0.23.1^20^ (parameters: --length_required 10000 --length_limit 30000) was used to filter the raw sequencing reads, with acceptable read lengths between 10 Kbp to 30 Kbp. Adapter-contaminated reads were removed using HiFiAdapterFilt^21^ with the default setting. Filtering and adapter removal resulted in 2,029,912 retained out of 2,038,748 raw reads (99.57%). The quality of the remaining reads was assessed with the default setting of FastQC version 0.11.5 (https://www.bioinformatics.babraham.ac.uk/projects/fastqc/), and the GC content graphs from the FastQC outputs were further investigated. GC content showed three peaks (Fig. S1a) (see figure deposited at Figshare)^22^. It is generally anticipated that the distribution of GC content follows a normal distribution close to the theoretical distribution. However, the peaks deviate from the theoretical normal distribution in this case. The first peak at ~35% and the second peak at 44.5% mean GC contents represent the GC content of nuclear and mitochondrial genomes, respectively^23,24^. Since the GC % of the chloroplast genome is similar to the nuclear genome, the third peak at ~51% mean GC content could be explained by contamination. Pbbm2 version 1.10.0 (https://github.com/PacificBiosciences/pbmm2), an SMRT minimap2 version 2.15^25^ (parameters:–unmapped–log-level INFO–log-file) wrapper was used to then map the raw reads to the cassava reference genome AM560-2 version 8^26^. BLAST search was conducted on unmapped reads to detect the presence of contaminants. BLAST results indicated that the third peak of the GC content originates from reads of *A. alternata*^27^. We further mapped the filtered reads to the *A. alternata* genome. A total 58,703 reads (2.89%) mapped with a consensus of 23.7 Mbp, roughly 69% of the *A. alternata* genome of 34.38 Mbp. The GC content of the *A. alternata* genome is 51%, confirming the third peak of the GC content plot. We extracted all the reads mapping to the *A. alternata* genome, resulting in 1,971,209 sequence reads with GC content having two peaks in the GC content plot, which we process as clean reads for the assembly (Fig. S1b) (see figure deposited at Figshare)^22^. Extracting mapped and unmapped reads was done using samtools v1.15.1 (parameters: samtools fastq -F 4 & -f 4)^28^.

### Genome assembly, scaffolding, and assembly quality check

Clean HiFi sequence reads of 45x estimated coverage with read length N50 of 17,513 bp (Table S1) (see table deposited in Figshare)^29^ were assembled *de novo* using hifiasm v0.16.1-r375 default settings, HiCanu v2.3 (parameters:-p out -pacbio-hifi genomeSize = 750 m -useGrid = false -merylThreads = 4 -merylMemory = 8 corOverlapper = ovl), and Flye v2.9.1-b1780 (parameters: --pacbio-hifi -o out --genome-size 750 m) assembly tools^13,30,31^. Utilizing outcomes from benchmarking analysis of these tools^11^ and outputs of these three assemblies, we opted to use the hifiasm assembly based on contiguity and achievement of haplotype resolved assemblies. Assembly statistics were compiled using QUAST^32^ default setting. The assembly comprised two haplotigs, hap1 and hap2. Before scaffolding, the two haplotigs assembly metrics showed hap1 and hap2 to consist of 362 contigs with a total length of 694 Mbp with a contig N50 length of 18 Mbp and 159 contigs with a total size of 665 Mbp with contig N50 length of 17 Mbp, respectively (Table 1). Scaffolding and ordering of the contigs were improved by RagTag^33^ (parameter: scaffold -o out -t 12) using TME204 reference, a chromosome-scaled genome^11^. The contiguity of the TMEB117 chromosome-scaled genome was improved, with hap1 N50 of 37 Mbp and hap2 N50 of 35 Mbp (Table 1). Eighteen pseudo-molecules representing the chromosomes were compiled for further annotation analysis. The unplaced contigs were separated from the sequences of chromosomes. These unplaced contigs of both haplotypes were then mapped to the chloroplast and mitochondrial genomes^23,24^. The alignment revealed a complete 100% coverage to the chloroplast genome and 90.39% and 62.63% coverage for hap1 and hap2, respectively, to the mitochondrial genome. These haplotigs provided representative sequences for the mitochondrial and chloroplast genome. The final scaffolded assembly of TMEB117 eighteen chromosomes was utilized for downstream analysis.

### Repeat landscape and gene annotation

**Fig. 2:**
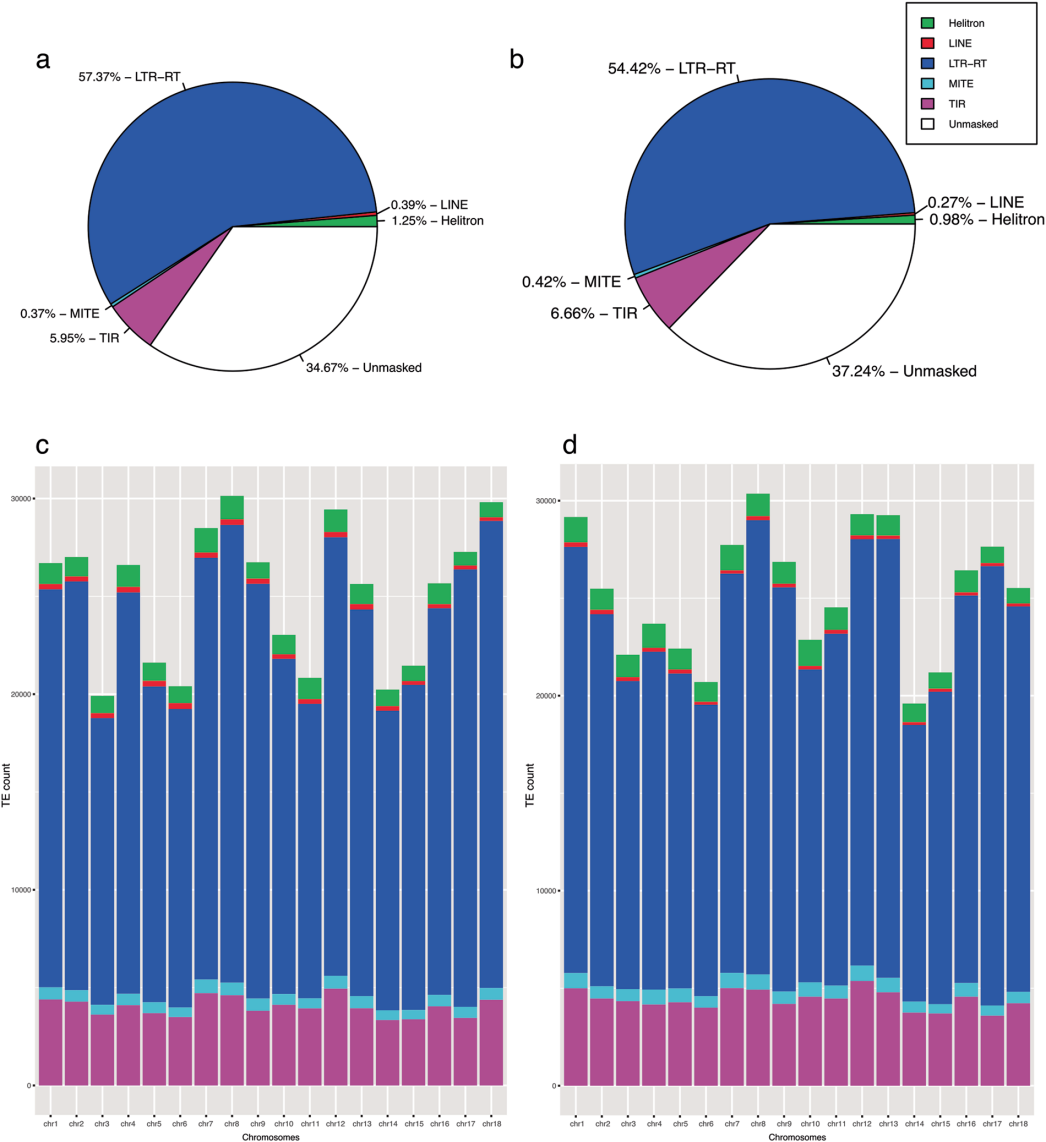
Illustrate the proportion and distribution of TEs across the chromosomes, as annotated by EDTA. (**a**) Shows the proportion of TEs identified in the hap1 genome, with the most abundant type LTR-RT (represented in the blue segment in the pie chart), covering 57.37% of the genome. (**b**) In hap2, LTR-RT remains the predominant TE family covering 55.07% of the genome. (**c**) Provides an overview of the distribution of TE families across all cassava chromosomes. (**d**) Slight difference in the distribution of TEs families annotated in the chromosomes of the hap2 genome compared to the hap1 genome.

The annotation of transposable elements (TE) was accomplished by using the Extensive *de novo* TE Annotator (EDTA)^34^ (parameters:–genome–overwrite 1–sensitive 1–anno 1–evaluate 1), combining structure and homology-based detection to identify predominant TEs in the assembled genome. The pipeline applies various tools, such as HelotronScanner, LTR_FINDER, LTRHarvest, LTR_retriever, TIR-Learner, RepeatModeler2, and RepeatMasker^35–41^, to classify novel TE sequences. We screened the outputs of EDTA using R and tidyverse package, resulting in non-redundant TE annotations and visualizations for both haplotypes. The generated repeat-masked genome was subsequently used for gene prediction. Consistent with other African cassava genomes^10,11^, over 50% of the genome constitutes repetitive elements. Specifically, in this study, 65.34% and 60.32% of the genome in hap1 and hap2 are transposable elements. The long terminal repeats – retrotransposons (LTR-RTs) are the most abundant, covering 57.37% (hap1) and 54.42% (hap2) of the genome size (Fig. 2a,b). *Gypsy* was the most abundant retrotransposons superfamily, occupying 41.14% (hap1) and 38.35% (hap2) of the genome (Table S2a,b) (see tables deposited at Figshare)^29^. The annotations are classified as families and superfamilies. Between the two haplotype genomes, there is a minimal difference in transposable element annotation percentage (Fig. 2a,b). However, the distribution of the TEs across the chromosomes differs between the two haplotypes (Fig. 2c,d). We used the Funannotate v1.8.9 singularity pipeline (see the script in the code availability section) to annotate the TMEB117 genome. The annotation pipeline involves three primary steps: genome masking, gene prediction, and functional annotation. Prior to annotation, the genome assembly as an output of EDTA annotation underwent soft-masked using scripts provided by the EDTA tool *make_masked.pl*. PASA alignment tool^42^ was used to generate an initial set of gene models by integrating RNA-seq data and protein homology to improve the accuracy of gene models. We used a set of 568,002 reviewed and curated protein sequences from a diverse array of species found in the UniProtKB/Swiss-Prot database release 2022_03 for the gene prediction step. Gene prediction was conducted using *ab initio* gene prediction tools, Augustus v3.3, SNAP v2013-02-16, and GlimmerHMM v3.0.4^43,44^ were employed for gene prediction. EVidenceModeler v1.1.1^45^ integrated gene models from various gene predictors and generated a consensus of the gene models. These gene models were used to generate protein sequences, which underwent filtering to remove proteins with less than 50 amino acids and to check for homology to transposable elements. The predicted genes were functionally annotated using the EggNOG database, UniProtKB, MEROPS, and CAZYmes^46–49^, giving insights into the biological functions and pathways. The resulting annotations were manually curated to correct errors and adjust gene models as necessary using the Funnannotate interface. Non-overlapping tRNA genes were predicted using tRNAscan-SE v2.0.9^50^. Transcript evidence was generated by Trinity v2.11.0^51^ through *de novo* transcript assembly, which was used in correcting, enhancing, and updating the predicted gene models. The haplotype-specific annotations for hap1 and hap2 genomes resulted in 47,138 and 49,163 genes, 53,264 and 55,222 transcripts, 836 and 814 tRNA, and 52,428 and 54,408 proteins, respectively. BUSCO analysis reveals 90% protein sequence completeness for both haplotypes.

### Orthologue analysis

**Fig. 3:**
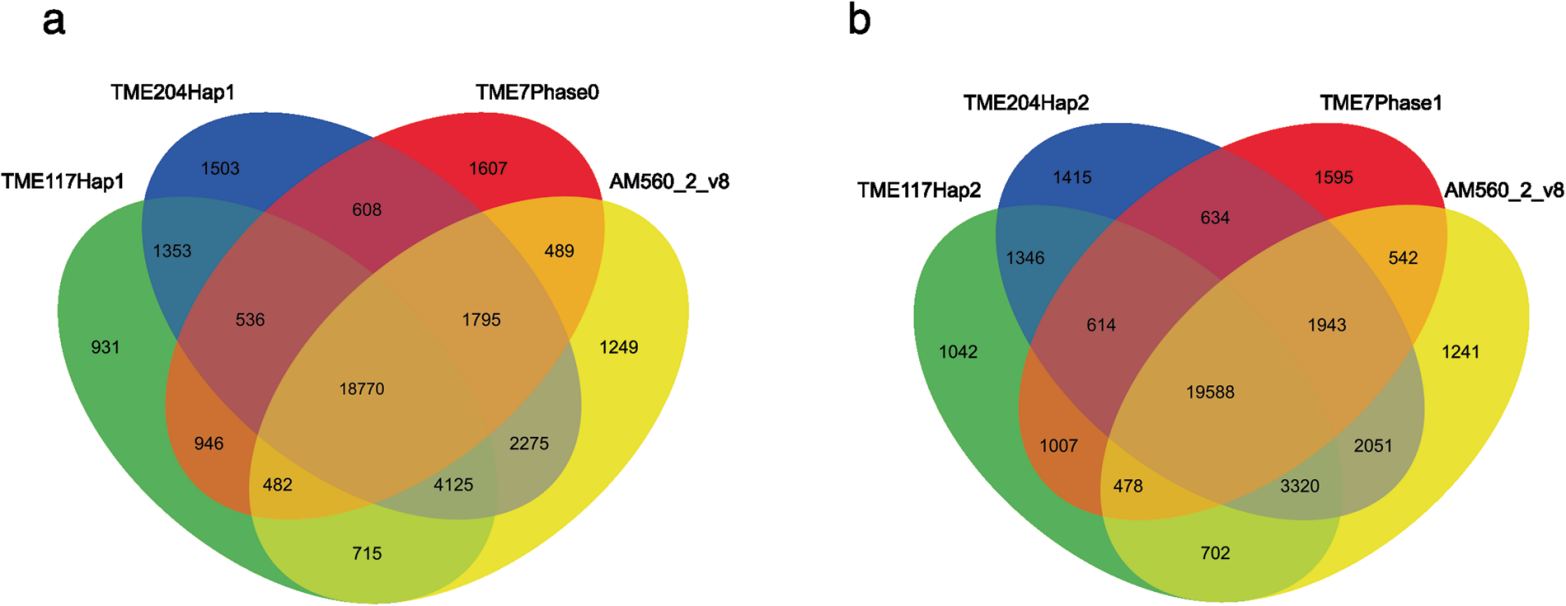
Venn diagram of the number of gene families shared among and unique to the haplotype genomes of three African cassava cultivars: TMEB117 (hap1 and hap2), TME204 (hap1 and hap2), TME7 (hap1 and hap2), in comparison to the reference genome AM560-2 v8. (**a**) 18,770 core gene families shared among the first haplotigs comparison with the reference genome AM560-2 v8. The second comparison (**b)**19,588 core genes on the second haplotig comparison with the reference genome AM560-2 v8. 931 gene families were unique in TMEB117 hap1 genome and 1042 in the hap2 genome.

The predicted protein sequences from the study were further analyzed by comparing them to other phased African cassava genomes, namely TMEB7 and TME204, with the AM560-2 v8.1 genome. Each haplotype was analyzed separately with the AM560-2 v8.1 genome. The OrthoVenn2 online tool (https://orthovenn2.bioinfotoolkits.net/) was used to identify orthologous protein groups across the genomes. The analysis was conducted with default parameters, including an inflation value of 1.5 for the Markov clustering algorithm and a BLASTP e-value of 1e-2. The resulting orthologous groups were visualized using the OrthoVenn2 web interface, which displayed Venn diagrams that indicate the number of unique and shared groups across the genomes. The OrthoVenn2 analysis identified 37,384 and 37,518 orthologous clusters, with 18,770 and 19,588 core genome orthologs for hap1 and hap2, respectively, indicating the presence of conserved groups across the genomes. In the TMEB117 hap1 and hap2 genomes, we observe fewer unique protein sequences (931 and 1042) than other cassava genomes, as shown in Fig. 3a,b.

## Data Records

Raw PacBio Hifi reads utilized for the assembly can be accessed from the National Center for Biotechnology Information (NCBI) Sequence Read Archive (SRA) database under BioProject PRJNA1002255, with accession numbers SRR25517176^52^ and SRR25517175^53^. The two chromosome-scaled haploid genomes have been submitted under distinct BioProject identifiers within NCBI, PRJNA1002865 for hap1 and PRJNA1002864 for hap2 with accession numbers JAWPHJ000000000^54^ and JAWPHK000000000^55^, respectively. The transcriptome data employed to annotate the genomes are available at NCBI with accession numbers SRR25537339^56^, SRR25537340^57^, and SRR25537338^58^. The genome annotation files are uploaded to Zenodo^59^.

## Technical Validation

**Fig. 4:**
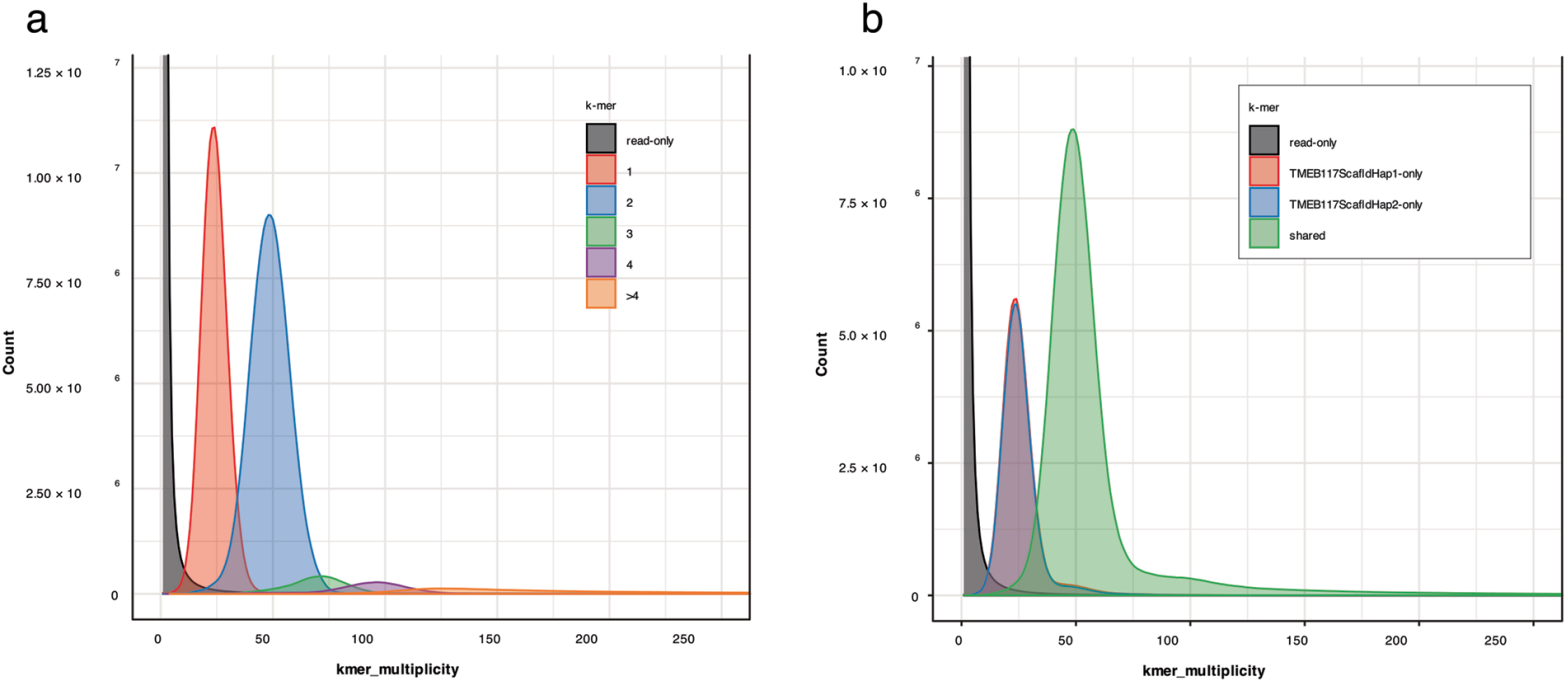
The completeness of resolved haplotypes assessed by Merqury copy number spectrum plots (**a**) and assembly plots (**b**). The x-axis represents the k-mer multiplicity, while the y-axis shows the abundance of k-mers. The grey region. represents the abundance of k-mers in the HiFi reads missing in the scaffold of the genome. (**a**) Copy number spectrum plot - the red peak observed at ~ 25x indicates heterozygous k-mers (1-copy k-mers), while the blue peak at ~ 50x represents the homozygous k-mers (2-copy k-mers). The other peaks show low levels of duplicated k-mers. (**b**) Assembly plot – k-mers coloured by their uniqueness, red peak (hap1), blue peak (hap2). At the heterozygous peak (25x), there is a slight difference in the k-mers indicating reconstruction of heterozygous variants was almost complete. Shared k-mers are shown in green which is at the 50x k-mer multiplicity.

The genome was validated by ensuring that the constructed assembly conforms with the data used to generate it. Over 99% of the raw sequence reads mapped to both the haplotigs. To evaluate the completeness, we used BUSCO v5.3.2 (parameters: -m genome eudicots_odb10) with orthologs from the eudicots lineage datasets, which included 2326 reference sets of genes specific to plants. BUSCO completeness score for the hap1 and hap2 assemblies was 98.9% (Fig. 1c). We employed Blobtools^60^ using the default setting to ascertain the absence of contamination by blasting the nucleotide database against the assembly and mapping the coverage of the assembly using HiFi reads to generate blobs. Blobs in the blob plot (Fig. S2) (see figure deposited at Figshare)^22^ were plotted at expected GC content percentages consistent with the GC plot after contamination removal. BLAST hits from the blob plot showed Streptophyta, which is a clade of plants. Therefore, we conclude that the assembly was free from contaminants. Haplotypic separation and assembly quality were achieved by performing a *k-mer*-based analysis using Merqury^61^ (*k-mer* = 21). The assembly has a quality value (QV) score of 64.38 for hap1 and 67.99 for hap2. The *k-mer* completeness for each haplotype assembly and the combined set was 78.63%, 77.95%, and 98.79%, respectively. This was approximately 20% of *k-mers* being haplotype-specific. So far, this genome is of better quality assembly, based on the QV score, compared to already assembled African cassava cultivars^10,11^ (Table 2). Figure 4 illustrates that the assembled sequence resulted in a nearly completely haplotype-resolved genome, as indicated by the copy number and assembly *k-mer* plots. Most heterozygous haplotype-specific *k-mers* were observed once in the assembled sequence, and the majority of homozygous *k-mers* were shared by the two genome haplotypes (Fig. 4a). In the heterozygous peak, slightly fewer *k-mers* differed between the two haplotypes (Fig. 4b), confirming that the reconstruction of heterozygous variants was almost thorough. We assessed the completeness of the predicted protein sequences within the eudicots lineage using BUSCO v5.3.2 (parameters: -m proteins eudicots_odb10) to validate the gene annotations. BUSCO analysis reveals 90% protein sequence completeness for both haplotypes (Fig. 1c). Subsequently, we performed a conditional reciprocal BLAST^62^, extracting the predicted gene model sequences from both haplotypes and compared them to the gene sequences of AM560-2, the cassava reference genome. Out of the predicted 47,138 genes in hap1 and 49,163 genes in hap2, we identified 30,456 in hap1 and 30,370 in hap2 reference gene sequences. Particularly, the reference AM560-2 genome, with a total of 32,805 genes, exhibited similarity to most predicted genes of both haplotypes of the TMEB117 genome.

**Table 2:** Quality value scores comparison table for TMEB117 and previously reported haplotype-resolved cassava genome assemblies.

	TMEB117	TME204	TME7
Primary assembly (hap1)	64.38	45.23	34.1
Alternative assembly (hap2)	67.99	48.94	34.4

## Data Availability

No custom programming or coding was used. Instead, the analysis utilized bash commands and the corresponding scripts stored within the GitHub repository accessible at: https://github.com/LandiMi2/GenomeAssemblyTMEB117.
